# Optimization of Thermophysical and Rheological Properties of Mxene Ionanofluids for Hybrid Solar Photovoltaic/Thermal Systems

**DOI:** 10.3390/nano11020320

**Published:** 2021-01-27

**Authors:** Balaji Bakthavatchalam, Khairul Habib, R. Saidur, Navid Aslfattahi, Syed Mohd Yahya, A. Rashedi, Taslima Khanam

**Affiliations:** 1 Department of Mechanical Engineering, Universiti Teknologi PETRONAS, Bandar Seri Iskandar 32610, Malaysia; balajibp1991@gmail.com; 2 Research Centre for Nano-Materials and Energy Technology (RCNMET), School of Science and Technology, Sunway University, Malaysia; saidur@sunway.edu.my; 3 Department of Engineering, Lancaster University, Lancaster LA1 4YW, UK; 4 Department of Mechanical Engineering, Faculty of Engineering, University of Malaya, Kuala Lumpur 50603, Malaysia; navid.fth87@siswa.um.edu.my; 5 Sustainable Energy and Acoustics Research Lab, Mechanical Engineering Department, Aligarh Muslim University, Aligarh 202002, India; smyahya.me@amu.ac.in; 6 College of Engineering, IT & Environment, Charles Darwin University, Ellengowan Drive, Casuarina, NT 0810, Australia; mabrur.rashedi@cdu.edu.au

**Keywords:** heat transfer fluid, ionic liquid, MXene, thermophysical, rheology, PV/T system

## Abstract

Since technology progresses, the need to optimize the thermal system’s heat transfer efficiency is continuously confronted by researchers. A primary constraint in the production of heat transfer fluids needed for ultra-high performance was its intrinsic poor heat transfer properties. MXene, a novel 2D nanoparticle possessing fascinating properties has emerged recently as a potential heat dissipative solute in nanofluids. In this research, 2D MXenes (Ti_3_C_2_) are synthesized via chemical etching and blended with a binary solution containing Diethylene Glycol (DEG) and ionic liquid (IL) to formulate stable nanofluids at concentrations of 0.1, 0.2, 0.3 and 0.4 wt%. Furthermore, the effect of different temperatures on the studied liquid’s thermophysical characteristics such as thermal conductivity, density, viscosity, specific heat capacity, thermal stability and the rheological property was experimentally conducted. A computational analysis was performed to evaluate the impact of ionic liquid-based 2D MXene nanofluid (Ti_3_C_2_/DEG+IL) in hybrid photovoltaic/thermal (PV/T) systems. A 3D numerical model is developed to evaluate the thermal efficiency, electrical efficiency, heat transfer coefficient, pumping power and temperature distribution. The simulations proved that the studied working fluid in the PV/T system results in an enhancement of thermal efficiency, electrical efficiency and heat transfer coefficient by 78.5%, 18.7% and 6%, respectively.

## 1. Introduction

**Figure 1:**
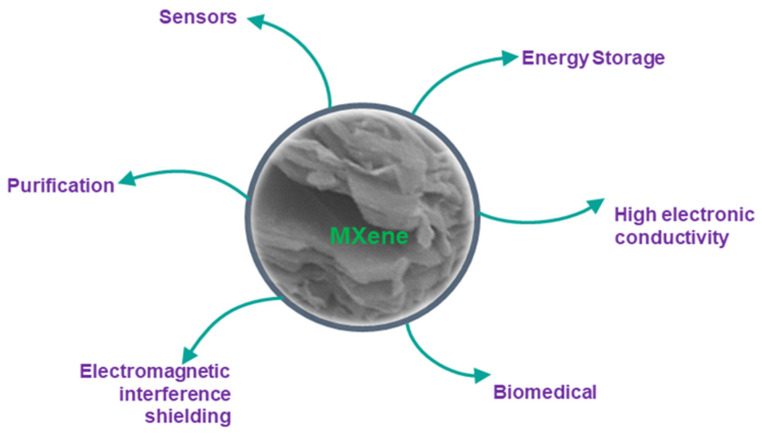
Prominent application of MXene nanoparticles.

In many industrial cooling and thermal transport applications, heat transfer fluid (HTF) is significant to enhance the thermal effectiveness of a system. Furthermore, in thermal systems, the traditional approach to increase heat dissipation rate is by increasing the heat transfer area of cooling devices and the flow velocity or dispersing solid nanoparticles in the heat transfer fluids known as nanofluids. Many researchers around the world have focused nanofluids as they provide the possibility of increased heat transfer for various purposes including cooling of thermal power plants, electronics, manufacturing and transportation. Most of the nanofluids’ works as heat transfer fluid (HTF) have concentrated on utilizing typical 2D nanoparticles such as graphene or conventional nanoparticles namely copper, silver, gold, aluminum oxide, copper oxide and silicon carbide [1,2,3,4]. For example, Wang et al. [5] showed a thermal conductivity enhancement of 14.2% using graphene nanoparticles in ethylene glycol base fluid. They also suggested that an increase in the nanoparticle concentration increases the nanofluid’s viscosity and thermal conductivity. Zhou et al. [6] investigated and compared the heat transfer capability of copper/Deionized water nanofluids with pure Deionized water on a miniature heat pipe with a mass concentration of 0.5 to 1.5 wt%. Authors found that copper nanofluid resulted in a better heat transfer capacity of 250 mW, whereas Deionized water obtained only 100 mW. Pourhoseini et al. [7] experimentally analyzed the heat transfer performance of a silver/water nanofluid in a plate heat exchanger considering the effect of volume flow rate and concentration. Authors reported that the tested nanofluid achieved a thermal conductivity enhancement of 36.6% than water. In contrast, nanofluid concentration has not impacted the system’s heat transfer coefficient than the volume flow rate. Loni et al. [8] presented an experimental study to evaluate the thermal performance of Al_2_O_3_/thermal oil, SiO_2_/thermal oil and pure thermal oil in a solar dish concentrator. Authors demonstrated that Al_2_O_3_ nanofluid attained the highest thermal efficiency than SiO_2_ nanofluid and pure thermal oil. These studies have conferred that several works have been performed to formulate heat transfer nanofluid with traditional nanoparticles due to their remarkable properties such as high thermal conductivity and stability, non-flammability, wetting, optimum viscosity, non-toxicity, minimum pressure drop, less corrosion and erosion. In 2011, Gogotsi from Drexel university discovered MXene for the first time and included it to the 2D materials community. MXene an innovative new class of two dimensional (2D) transition metal carbide nanoparticle possessing phenomenal surface area, morphology, conductivity, cyclic stability, tunable surface groups fascinated present researchers to utilize it in the field of heat transfer enhancement. Furthermore, in recent research, MXene has played an essential role in environmental uses such as reducing gases, organic materials, heavy metals and radionuclides [9,10]. As a result of their non-toxic nature, most biomedical applications focused on Ti-, Nb- and Ta-based MXenes. Jastrzębska et al. [11] researched the impact of surface oxidation layers and nanoparticles on 2D Ti_3_C_2_ MXene sheets’ surface. Authors found that delaminated MXene can kill cancerous cells and proved that high concentration (>375 mg·L^−1^) of MXenes is more toxic. Nasrallah et al. [12] examined MXene’s biocompatibility by evaluating their possible toxicity using the zebrafish embryo model. Authors concluded that low concentration of MXenes (<100 μg/mL) is safe and recommended for potential applications in water treatment and environmental remediation. The main aspects of MXene in different applications are presented in Figure 1. 2D nanomaterials are rarely discussed, even though many potential high value-added applications are summarized in Table 1. This article intends to raise the enhancement approaches of MXene-based heat transfer fluid, especially on thermal and rheological efficiency.

Other than water, glycol and oils nanoparticles can be added to ionic liquids for obtaining a unique form of fluids called ionic liquid-based nanofluids or ionanofluids which have a greater potential for next-generation heat transfer fluids especially for applications with high temperature [20]. Water and glycol-based nanofluids are not appropriate for medium to high-temperature use while thermal oils undergo flammability above 250 °C. Consequently, new nanofluids based on fluids other than those used in traditional fluids are required for medium to high-temperature applications. Ionic liquids have attracted many researchers due to its high thermal stability, high dissolution capacity, high conductivity, non-volatility, non-toxicity and low melting point. Murshed et al. [21] investigated the effect of ionanofluids ([C4 mim][NTf2]+MWCNT) in a heat exchanger. They proved that ionanofluids exhibit superior thermal properties and require only a small heat transfer area than their base ionic liquids. Oster et al. [22] investigated the thermal conductivity of different ionanofluids and ionic liquids. Anion was an important element affecting thermal conductivity, with bulkier anions contributing to higher enhancement values. Recently, Oster et al. [23] used [P14,6,6,6][RO] cations with acetate, butanoate, hexanoate or octanoate anion blended in carbon nanotubes, boron, nitride or graphite and characterized its thermophysical properties. The presence of nanoparticles in ionic fluids contributed to positive heat capacity, thermal conductivity and density. However, such an addition induced a substantial increase in viscosity that affects the other properties like pumping power, surface tension etc. Another criterion, nanoparticle morphology, presented by Jozwiak et al. [24] analyzed the enhancement of thermal conductivity in carbon-based ionanofluids. With increased aspect ratio and surface area of the dispersed carbon nanotubes, the thermal characteristics of the ionanofluids showed a substantial increase. In a different study, Vallejo et al. [25] analyzed the tribological performance of imidazolium-based ionanofluids ([Emim][MS]/Silicon nitride) with 0.1 to 1 wt% mass concentration. At 0.25 wt% mass concentration, ionanofluids obtained the maximum reduction in the friction coefficient with less wear volume. Jozwiak et al. [26] took a comprehensive overview of the rheological significance during ionanofluid flow. Valkenburg et al. [27] demonstrated that [Emim][BF_4_], [Bmim][BF_4_] and [Dmpi][FSI] ionic liquids were thermally stable at 450 °C. Paul et al. [28] improved the thermal performance of Al_2_O_3_ nanofluids using 1-butyl-3-methylimidazolium bis trifluoromethyl sulfonyl imide ([C_4_mim][NTf_2_]) ionic liquid in a circular tube. For a certain range of Reynolds number, ionic liquid-based nanofluid’s heat transfer coefficient achieved a significant improvement. Xie et al. [29] used 1-ethyl-3-methylimidazolium diethyl phosphate ([Emim][DEP]) ionic liquid on MWCNT/Water nanofluid to evaluate its thermal conductivity, viscosity and density. Their results illustrated a maximum thermal conductivity enhancement of 9.7% than the base fluid (water) whereas density and viscosity showed a linear temperature dependence like conventional nanofluids. Minea et al. [30] numerically evaluated the thermophysical properties of Al_2_O_3_/([C_4_mim][NTf_2_]) ionanofluids with 0.5, 1 and 2.5 vol% mass concentrations under natural convection mode considering Rayleigh number (104 ≤ Ra ≤ 106) and volume concentration (0% ≤ *ϕ* ≤ 2.5%) aspects. They found that the ionic liquid-based nanofluids’ thermal efficiency was mainly affected by the buoyancy and viscous force. In addition to the thermal property, viscosity is also an essential property of fluids, especially for its practical application in fluid flow and heat transfer. Furthermore, the perception of convective heat transfers and pumping power is directly linked to viscosity in flow systems. Soman et al. [31] discussed the effect of viscosity on the heat transfer performance of ionic liquid ([Bmim][Br])/water and nanoparticle (γ-Al_2_O_3_)/water fluids. These researchers observed that ionic liquid increased the absorption of energy from hot fluid and decreased viscosity at higher temperature influenced the turbulent characteristics of fluid flow that led to the heat transfer rate enhancement. In an interesting study, Cao et al. [32] demonstrated that ionanofluid (Imidazolium ionic liquids/Fe_2_O_3_) stability increased with increased viscosity, but it made a huge impact on decreasing the absorption properties. The recent growth of nanofluids’ publications demonstrated that thermal conductivity and specific heat capacity had been paid much attention than viscosity. In addition, research findings indicated that nanofluid viscosity was very high compared with their base fluids and with increasing concentration of nanoparticle it further increases, which indirectly affects the pumping power and pressure drop of the fluid flow. In order to exploit their potential in the broader field of applications apart from nanoparticle concentration, it is important to study the impacts on the viscosity of nanofluids from other factors like temperature, Newtonian and non-Newtonian behavior, base fluids, dispersion, particle type, size and shape in which this study intends to do.

Majority of articles published concentrated on different properties of conventional nanoparticle-based nanofluids, and only a few researchers investigated all the thermal and rheological properties. On the other hand, development of novel nanofluid consisting of 2D MXene induced by 1-ethyl-3-methylimidazolium ([C_8_SO_4_]) ionic liquid with high thermal conductivity and dispersion stability has not been reported which motivates the present experimental investigation. This research’s main objective was to analyze the thermophysical and rheological properties of ionic liquid-based MXene nanofluid with a mass concentration of 0 wt% to 0.4 wt% at different temperature range and its performance with PV/T systems. The heat transfer efficiency of the solution containing MXene+[C_8_SO_4_]+DEG in turbulent conditions is calculated using theoretical correlations. Based on the experimental values, a numerical model is conferred to analyze the potential of using ionic liquids in nanofluids for PV/T systems. The proposed model assesses and compares the thermal, electrical and heat transfer coefficient of a PV/T system using ionic liquid-based 2D MXene nanofluid at various concentrations and mass flow rates.

## 2. Materials and Methods

### 2.1. Materials

1-ethyl-3-methylimidazolium octyl sulfate ([C_8_SO_4_]) (CAS-NO: 790663-79-5) ionic liquid (purity ≥ 98%, halides ≤ 0.1%, water ≤ 1%, molar mass of 320.45 g/mol) and diethylene glycol with a molar mass of 106.12 g/mol (CAS-NO: 111-46-6) was purchased from Merck group, Darmstadt, Germany. MXene (Ti_3_C_2_) nanoparticle was synthesized in the lab (RCNMET, Sunway University, Kuala Lumpur, Malaysia) through the procedure followed in one of our previous literatures [33].

### 2.2. Methods

**Figure 2:**
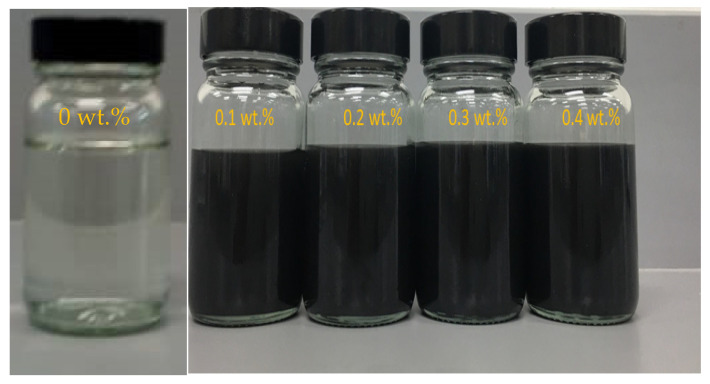
Raw image of the prepared ionic liquid-based MXene nanofluid.

In the ionic liquid and diethylene glycol solution, the synthesized dry MXene nanoparticles have been blended with a magnetic stirrer for 30 min (40 °C, 1000 rpm) and sonicated with an ultrasonic processor (750 W, 20 kHz, Sonics VCX-750, Newtown, CT, USA) of 60% power and 30% amplitude for 90 min to obtain a homogeneous dispersion [34,35,36]. PULSE mode is chosen with 20 s ON cycle and 10 s OFF cycle. A two-step method is more economical and easy; it was followed for preparing all the samples [37,38]. Due to the agglomerates’ vibrations, which was caused by the ultrasonic waves, the temperature of the sample increased rapidly. In order to overcome this issue, a water bath has been incorporated with the ultrasonic processor to maintain the temperature of the sample at a constant rate. The prepared samples’ different proportions were as follows: [C8SO4]+DEG+MXene with a mass concentration of MXene of 0 wt%, 0.1 wt%, 0.2 wt%, 0.3 wt% and 0.4 wt% and the ratio of ionic liquid and diethylene glycol mixture was 1:9. The quantity of nanoparticles added to ionic liquids and diethylene glycol is shown in Table 2. Figure 2 shows the formulated nanofluids’ photo with 0, 0.1, 0.2, 0.3 and 0.4 wt% MXene without surfactant addition and its dispersion stability was investigated by visual inspection method.

### 2.3. Characterization

At a temperature of 20 to 80 °C, the prepared samples’ thermal conductivity was evaluated using a thermal property analyzer (Tempos, METER Environment, Pullman, WA, USA). This device accomplished with ASTM 5334 standard could work under a temperature range of −50 to 150 °C with a 6 cm length KS-3 sensor having a maximum thermal conductivity testing capacity of 0 to 2 W/m·K. The samples’ temperature was controlled by a water bath (WNB22, MEMMERT, Büchenbach, Germany), and the volume of each sample used for testing was around 30 mL. For accuracy, the measurement was performed three times at each temperature, and the mean values were reported in this paper.

The sample’s density was measured by a density meter (DMA 1001, Anton Paar, Graz, Austria) at a temperature ranging from 20 to 60 °C that works on the principle of pulsed excitation method. For certainty, the density meter was calibrated with distilled water. The specifications of DMA 1001 are as follows: density measuring range of 0 g/cm^3^ to 3 g/cm^3^; pressure range of 0 psi to 145 psi; temperature range of 15 °C to 60 °C; minimum sample volume of ~1 mL. For accuracy, the measurement was performed two times at each temperature, and their mean values were reported in this paper.

Differential scanning calorimeter (PerkinElmer, DSC 4000, Waltham, MA, USA) with temperatures ranging from 20 °C and 80 °C was used to measure the sample’s specific heat capacity. In this experiment, samples are heated, and their specific heat capacity is measured with respect to heat flow. DSC is mainly used to evaluate how a substance’s heat capacity is changed by temperature.

The samples’ rheology and viscosity were measured using a Rheometer (MCR 92, Anton Paar), which has an air-bearing motor that provides measurement in both oscillation and rotation mode has a maximum torque of 125 m·Nm. The specifications are as follows: maximum temperature range of −40 to 400 °C; maximum speed of 1500 rpm; the maximum angular velocity of 157 rad/s; maximum angular frequency of 628 rad/s.

MXene nanoparticles’ morphology was analyzed using Field Emission Scanning Electron Microscope (Zeiss Supra 55 VP, Oberkochen, Germany). Thermogravimetric Analysis (PerkinElmer TGA 4000) was used for determining the thermal stability of the as-prepared ionic liquid-based nanofluids. FTIR (Fourier Transform Infrared) spectroscopy is one of the most useful methods to find the molecular groups on the as-prepared samples. Perkin Elmer Spectrum Two FTIR Spectrometer was used in this study where infrared radiation is absorbed by the prepared samples which caused specific vibrations in functional groups, creating an infrared spectrum fingerprint of the sample.

Raman Spectroscopy can give detailed information about a sample’s structure during the interaction of light with matter. It is one of the simplest, quickest and most useful techniques to determine the chemical, structural and electronic information on different materials. UV Raman Spectrometer (Horiba Jobin Yvon HR800, Kyoto, Japan) equipped with confocal LabRAM microprobe and triple spectrometer design for unprecedented stable light was used in this study. The triple additive configuration of the HR800 system will be advantageous to find the Raman bands’ location with very high resolution.

### 2.4. Uncertainty Analysis

In order to ensure the reliability of experimental measurements and to determine the sensitivity of uncertainty levels for various quantities depending on their relevance to measurements, uncertainty analysis is performed. The uncertainty of a quantity (*u*) is calculated by the following Equation (1).

(1) $$u=\frac{S}{\sqrt{n}}$$

where *S* is the calculated standard deviation and *n* is the number of measurements in the set. The uncertainty of the measured quantities such as thermal conductivity, viscosity, density, specific heat capacity, thermal stability and light transmittance (FTIR spectroscopy) is depicted in Table 3. The uncertainty calculation for each measured quantity is provided in the Supplementary Material. For all cases considered in this research based on the above equation, the measurement of uncertainty is less than 1%. This is a reliability indication of the test data assessed.

## 3. Results and Discussion

This section explores the thermophysical and rheological properties of the formulated samples at different parameters. In addition, these properties are dependent on the dispersion stability of nanoparticles in base fluids. Therefore, the stability of the prepared ionanofluid is evaluated using a visual inspection method and Spectral absorbance method, which is briefly explained in our previous literature [38]. The results showed that the synthesized ionanofluids are highly stable for about 7 days.

### 3.1. Structural and Morphological Analysis of MXene Nanoparticles

**Figure 3:**
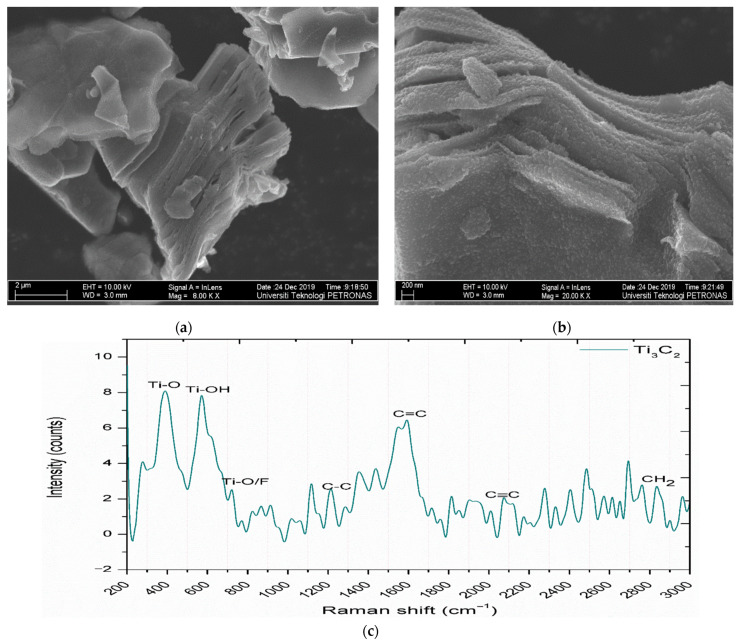
(**a**,**b**) FESEM image of the synthesized MXene nanoparticle at 8000× and 20,000× magnification, (**c**) Raman spectrum collected on the surface of the MXene nanoparticle.

One of the most popular and widely used techniques for analyzing the topography, morphology, composition and crystallographic information of nanoparticle is FESEM. The Field-Emission Scanning Electron Microscope (FESEM) uses a field-emission cathode to provide high and low energy straight rays, thereby improving the spatial precision and lowering the sample charging and loss. High quality, less distorted and low voltage images could be obtained with negligible electric charging varying from 0.5 to 30 kV. The FESEM image of the synthesized MXene nanoparticle is shown in Figure 3a,b. The FESEM image confirms the presence of multilayers in the as-prepared MXene nanoparticles.

Photons are elastically diffused when light is scattered from a molecule. Raman effect is defined as the process that leads to this inelastic light scattering which will be almost one in 107 photons. The difference in the frequency of the incident beam light and scattered radiation is known as Raman shift, which provides information on matter’s vibrational properties. The Raman Spectrum of Figure 3c shows the formation of Ti_3_C_2_, which is consistent with the previous study [39]. In addition to the characteristic broad peak at 1600 cm^−1^, two sharp peaks having a maximum at 400 cm^−1^ and 600 cm^−1^ were observed, commonly attributed to E_g_ vibrations that illustrate Ti-O’s presence Ti-OH groups. The appearance of small peaks from 1200 cm^−1^ to 2100 cm^−1^ highlighted the fact that carbon groups are ubiquitous surface groups of MXene.

### 3.2. Thermal Conductivity

**Figure 4:**
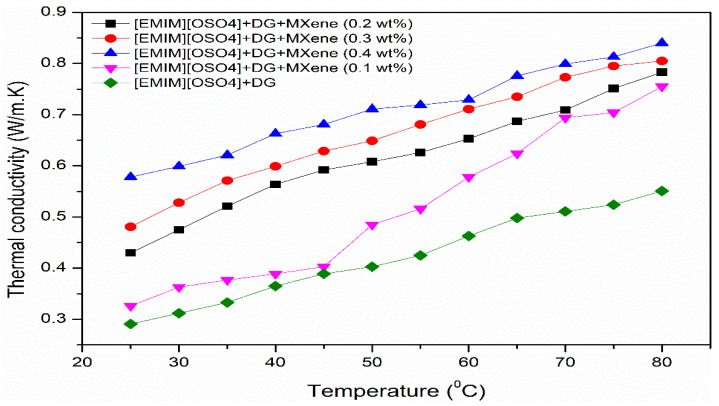
Temperature dependent thermal conductivity of the prepared ionanofluids.

Thermal conductivity is a measure of a medium’s capability to conduct heat that is mostly dependent on the type of material and temperature. In addition, it is one of the essential properties in many industrial and consumer applications. This section investigates the impact of temperature in the as-prepared samples ([C_8_SO_4_]+DEG+MXene) on its thermal conductivity using Tempos thermal property analyzer at a temperature ranging from 25 to 80 °C with a mass concentration of 0 to 0.4 wt%. Figure 4 shows the experimentally measured thermal conductivity values of the as-prepared samples. As expected, formulated ionic liquid-based nanofluids’ thermal conductivity increased with a rise in temperature and concentration, which is consistent with previous studies [40]. The possible mechanisms responsible for enhancing thermal conductivity include Brownian motion, aggregation of nanoparticles and molecular level liquid layering of the nanoparticles-liquid interface. MXene nanoparticles in the prepared ionanofluids were attributed to random thermal motion through particle-particle collision caused by Brownian movement during heating which increased the thermal conductivity of the as-prepared ionanofluid. On the other hand, the high order arrangement made by the surrounding liquid layers of nanoparticles will increase the free mean path in the ionanofluid through which phonons can move rapidly, leading to thermal conductivity rise. Nanoparticles are often agglomerated when dissolved in liquids, which is considered one of nanofluids’ contentious heat transfer mechanisms. The aggregation of nanoparticles into percolating forms normally induces thermal resistance, greatly influencing the overall thermal conductivity. Therefore, the utmost care must be given to prevent agglomeration or sedimentation of nanoparticles in base fluids.

From the results, it was found that low concentration of ionic liquids with MXene/DEG nanofluid contributed to the enhancement of thermal conductivity which can be attributed to the interionic interactions of [C_8_SO_4_] ionic liquid at a molecular level linked with 2D MXene nanoparticle structure resulted in good stability and thermal conductivity. The other factor for superior thermal conductivity is the excellent thermal property of 2D MXene nanoparticle. Ultimately, the uniform distribution of high viscous [C_8_SO_4_] ionic liquid over the MXene nanoparticle prevented the agglomeration rate and enhanced the active surface area for conducting a high quantity of heat. Furthermore, the thermal dissipation usually occurs on the particle surface, so nanoparticles with a large surface area were more advantageous. At an average temperature of 60 °C, the enhancement of thermal conductivity rises with increasing MXene nanoparticle mass concentration, from 24.83% for 0.1 wt% to 57.45% for 0.4 wt%.

### 3.3. Density

**Figure 5:**
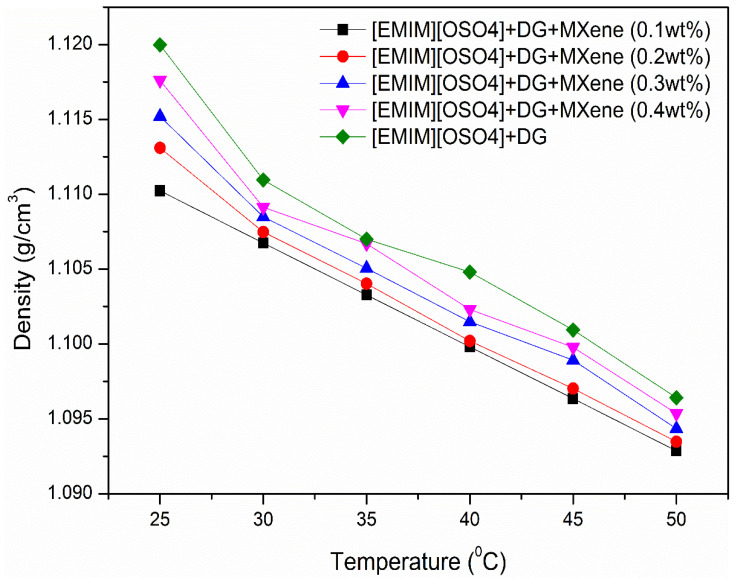
Variation of density with respect to temperature for the prepared ionanofluids.

Another vital property that defines the efficiency of ionanofluids in heat transfer application is density. In fact, the pumping power, pressure drop, separation of liquid–liquid phase and mass transfer play a significant part in fluid flow applications dependent on the effective density. In addition, Reynolds number is mainly based on the fluid density and therefore, for calculating the heat transfer coefficient, an accurate estimation of density is essential. Figure 5 shows the temperature dependence of the tested ionic liquid-based nanofluids. From the results, the density of the as-prepared samples decreased with an increase in temperature, which was in good agreement with other studies [41,42]. On the other hand, the density of the prepared ionic liquid-based nanofluids increased with nanoparticles’ addition as MXene has high inherent density. Limited literature of MXene based fluids does not permit us to give an in-depth explanation of this property.

### 3.4. Viscosity

**Figure 6:**
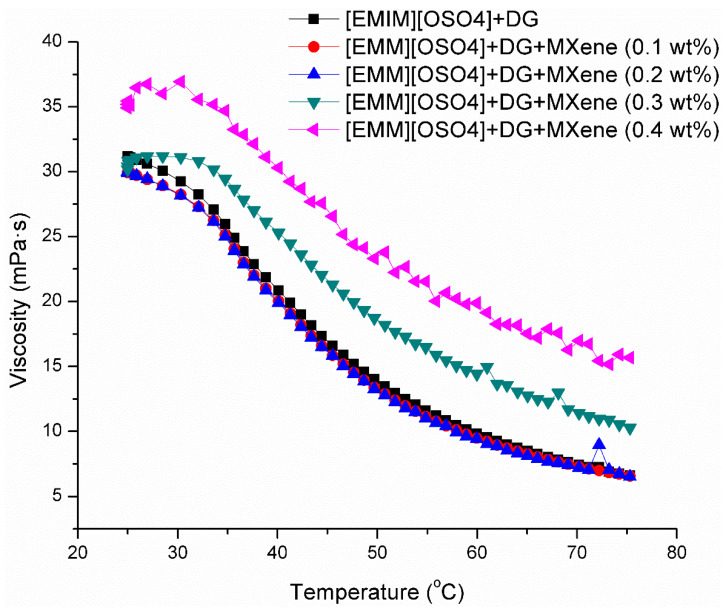
Viscosity of the prepared ionanofluids at different temperatures.

Viscosity is a fundamental property of liquids where the internal resistance of a flow can be measured with respect to the temperature, pressure and nanoparticle concentration. A broad range of engineering problems exhibiting momentum transfer and fluid flow can be solved by having adequate viscosity knowledge. With increasing temperatures, the bonds responsible for cohesion are broken attributing to the decrease of liquid’s viscosity. Generally, nanofluid’s viscosity is considerably higher than the base fluids and increases further with the addition of nanoparticles and ionic liquids. Figure 6 shows the viscosity as a function of temperature for the as-prepared samples. According to the obtained results, with the increase in temperature from 25 to 75 °C, the formulated ionic liquid-based nanofluids’ viscosity decreased from 38 mPa·s to 6 mPa·s at MXene nanoparticle concentration ranging from 0 to 0.4 wt% and with a constant shear stress of 1.5 Pa. Moreover, the samples’ viscosity with a nanoparticle concentration of less than 0.2 wt% has not revealed any significant changes over the concentration increase of MXene nanoparticles. From the flow behavior of ionanofluids, the pattern of viscosity with changes in the applied shear stress is very significant. The variation of ionanofluid viscosity with the increased shear rate at 25 °C, 50 °C and 75 °C is presented in Appendix A. Due to the increased shear stress, exponential decrease in viscosity occurred, which confirmed shear thinning behavior in all studied temperatures. Therefore, it can be concluded that the formulated ionanofluids prove to be a non-Newtonian pseudoplastic nature. It can also be noted that the viscosity is higher at a low shear rate that may be due to the irregular arrangement of nanoparticle offering high flow resistance at the beginning. After a certain time, these nanoparticles get arranged with increased shear rate leading to reduced viscosity. This is because molecules break down at higher shear rates and get perfectly aligned.

**Figure 7:**
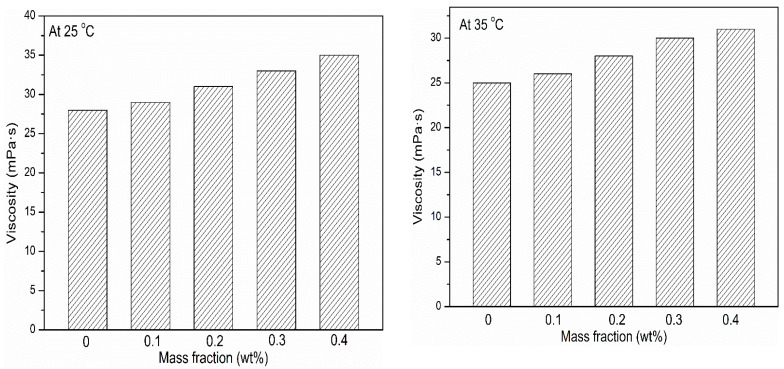

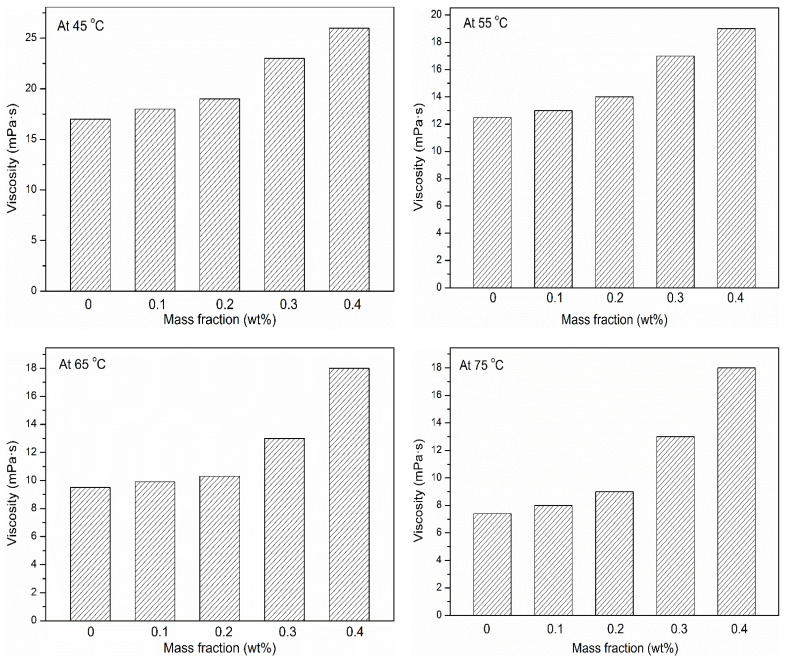
Viscosity of the ionanofluids over mass fraction at different temperatures.

Furthermore, the pumping power, which is considered an important factor in dissipating the provided heat load for different types of refrigerants, depends on the viscosity of the fluid [43]. Effective heat transfer fluids or coolants should handle the same heat load using less fluid and less pumping power. Moreover, heat transfer coefficients in turbulent flow can be increased by increasing the pumping power and flow rate. In the same way, more pumping power and velocity is required for maintaining Reynold’s number of a highly viscous fluid. The effect of the mass fraction on the viscosity of the ionanofluid is presented in Figure 7. In general, the addition of nanoparticles to ionic liquids increases the viscosity of the ionanofluid, consistent with the present study. The obtained results demonstrate that increase in the nanoparticle concentration leads to viscosity increase. Furthermore, the viscosity enhancement of the nanofluids is calculated by Equation (2).

(2) $$\eta_{Enh}=\frac{100\eta_{INF}}{\eta_{IL}-100}$$

where *η_Enh_* indicates the viscosity enhancement, *η_IN_*_F_ represents the viscosity of the tested ionanofluids and *η_IL_* depicts the base ionic liquid. The viscosity of the base ionic liquid is acquired from Costa et al. [44]. Furthermore, the enhancement of viscosity was found to increase with temperature in the ranges of 5.58–6.98% at 25 °C, 11.41–14.15% at 35 °C, 20.14–30.8% at 45 °C, 89.2–135.7% at 55 °C, 94.31–158.12% at 65 °C, and 101–171.85% at 75 °C. This proves that viscosity enhancement is linearly dependent on the temperature.

### 3.5. Rheological Analysis

Rheological characterization is also of great importance, for the practical applications of fluid flow and heat transfer, especially for determining the pumping power and convective heat transfer of ionic liquid-based nanofluids. It can also be considered as a drag force and represents the frictional characteristics of the fluid. Moreover, viscosity plays a significant role in determining the power requirement of pumps, pipeline configurations, transportation and storage. ANTON PAAR rotational Rheometer (MCR 92) was used to determine the viscosity of MXene-based ionanofluid at various temperatures (25–75 °C) and shear levels (30–100 S^−1^) and with certain nanoparticle concentration (0, 0.1, 0.2, 0.3, 0.4 wt%). Generally, the viscosity of a fluid is unchanged regardless of the shear stress applied. For fluid flow thermal systems, this viscosity reduction is ideal for optimal efficiency. We infer that the flow results in the rise and fall of liquid surface levels without any yield stress for dilute solutions and suspensions even for little stress. For more concentrated solutions, particularly for 0.3 wt% and 0.4 wt%, there is understandable doubt whether yield stress exists or not.

Rheology is one of the main characteristics which defines the flow and deformation of fluids under different mechanical forces. When subjected to flow, deformation or stress, each substance exhibits its behavior. In many applications such as inkjet printing, protein formulations/injections, food/drink manufactures, a strong understanding of rheological characteristics such as shear tension, shear pressure, shear rate, distance, materials, base fluid, temperature, time, surfactants, shape and concentration of nanoparticles is essential. Moreover, rheology provides an understanding of flow characteristics critical for determining the required pressure drop and pumping power. Newtonian fluids are fluids where the shear stress is directly proportional to the shear rate, and the molecules do not possess significant anisotropy.

**Figure 8:**
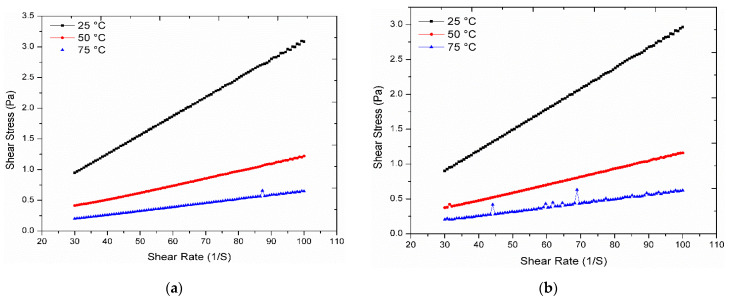

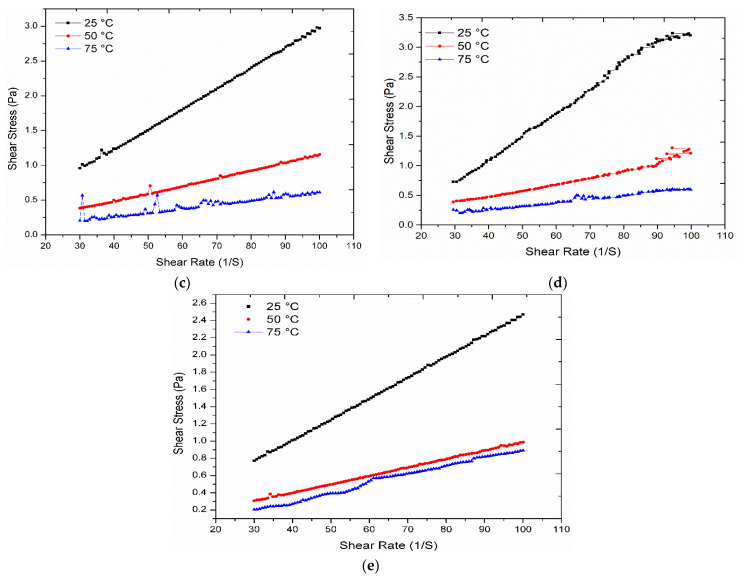
Shear stress as a function of shear rate for (**a**) [C_8_SO_4_]+DEG, (**b**) [C_8_SO_4_]+DEG+ 0.1 wt% MXene, (**c**) [C_8_SO_4_]+DEG+ 0.2 wt% MXene, (**d**) [C_8_SO_4_]+DEG+ 0.3 wt% MXene, (**e**) [C_8_SO_4_]+DEG+ 0.4 wt% MXene.

Figure 8 demonstrates the shear rate and shear stress for 0 wt%, 0.1 wt%, 0.2 wt%, 0.3 wt% and 0.4 wt% at various temperatures of 25 °C, 50 °C and 75 °C for the as-prepared ionanofluids. The solid line illustrates the relationship between the shear rate and shear stress in the graphs. The effect of shear stress on shear rate has been observed to be steeper by decreasing the temperature that corresponds to the standard Newtonian model. From the results, it was clear that shear stress improved with an increase in shear rate and decrease in temperature except for 0.3 wt% (Figure 8d) where shear stress at 75 °C and 50 °C were high while 25 °C showed less shear stress which was contrary to other samples, the reason may be due to the improper dispersion of nanoparticles. It was also observed that the maximum shear stress was obtained for low concentration, and it continued to decrease with increase in MXene addition. For example, ionanofluid with 0, 0.1, 0.2, 0.3 and 0.4 wt% MXene produced maximum shear stress of 3.4, 3, 2.9, 3.3 and 2.5 Pa, respectively. The findings revealed that all the studied samples showed approximately a linear pattern of shear stress with different shear rates at 25 °C, 50 °C and 75 °C. This shows clearly that the formulated ionanofluids possess Newtonian characteristics at different mass concentration of MXene nanoparticles. The gradient rise of the line in each diagram is due to the influence of temperatures on the nanofluid’s viscosity. On the four as-prepared samples, it is found that the slope of the line at 25 °C is considerably greater than the slope of the line at 75 °C. Overall, these results demonstrate that nanoparticle concentration and temperature are the major factors in nanofluid’s rheology.

### 3.6. Thermal Stability

**Figure 9:**
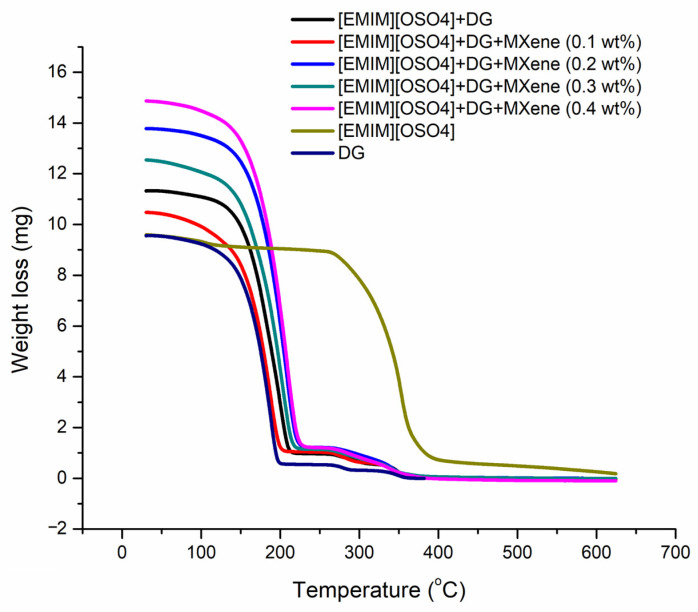
Comparison of thermal stability of the prepared ionanofluids with pure [C_8_SO_4_] and diethylene glycol.

The thermal degradation due to high heat conditions in thermal systems is one of the main reasons for the failure of heat transfer and lubrication fluids. In order to overcome this, thermal analysis was done to determine the thermal decomposition of the as-prepared samples. As such, the as-prepared samples were subjected to Thermogravimetric analysis (TGA) from 30 °C to 650 °C with a heating rate of 10 °C/min under 20 mL/min flow rate of nitrogen gas. The five as-prepared samples’ thermal decomposition is presented in Figure 9 with different content of MXene nanoparticles, namely 0 wt%, 0.1 wt%, 0.2 wt%, 0.3 wt% and 0.4 wt%. All of the four concentrations of MXene improved the initial decomposition temperature with constant weight loss. The MXene ionanofluids obtained a higher degradation temperature with 0.4 wt% MXene. The measured degradation temperature (i.e.,) the temperature at which the weight loss begins (T_onset_) was 172.74 °C, 166.27 °C, 182.26 °C, 167.89°C and 172.89 °C with a weight loss of 88.466%, 83.631%, 83.641%, 87.307%, 89.166% when the content of the as-prepared samples contained 0, 0.1, 0.2, 0.3 and 0.4 wt% MXene nanoparticles, respectively. The first derivative peak temperatures, i.e., the point of the greatest change rate on the weight loss curve for the as-prepared ionanofluids, were 200, 187.24, 207.40, 202.15 and 208.72 °C which are also known as inflexion points. The results showed that the addition of MXene nanoparticles improved the thermal stability of the as-prepared ionanofluids with an average weight loss of 17%/min and the thermal stability of the sample showed complete degradation after 200 °C.

The [C_8_SO_4_] ionic liquid showed maximum thermal stability of 316 °C while pure diethylene glycol started to degrade from 162 °C. As a result, the onset temperature (Tonset) for the tested ionic liquids and diethylene glycol was measured to be 316 °C and 162.01 °C while the first derivative point (Tp) was 352.45 °C and 187.09 °C, respectively. As discussed above, the as-prepared samples’ thermal stability improved when compared with the thermal decomposition temperature of diethylene glycol. From the graph, it was clear that the thermal decomposition of all the studied fluids occurred between 160 °C and 200 °C except ionic liquid. The ionic liquid was thermally stable at high temperature due to strong covalent bonds between the molecules that cannot be broken easily, leading to high decomposition temperature thresholds. This study recommends increasing the proportion of ionic liquids with base fluids to enhance the thermal stability of ionanofluids further.

**Figure 10:**
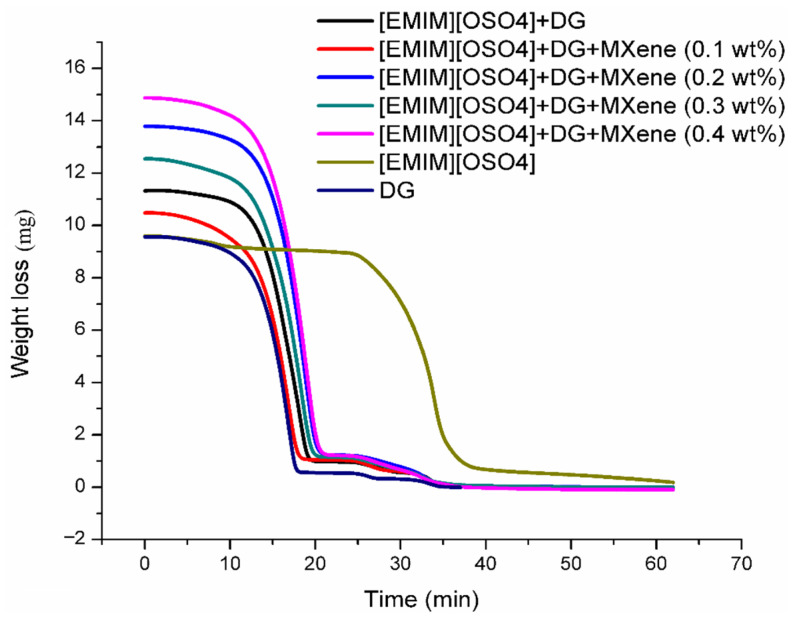
Loss of mass with respect to time at 200 °C.

Thermogravimetric Analysis determined the upper use time of the as-prepared samples. Beyond this specific time, the samples will begin to degrade. Figure 10 depicts the loss of mass as a function of time for the prepared samples measured by Thermogravimetric analysis. After 20 min and around 200 °C, significant decomposition is evident with an average decomposition rate of ~17% per minute. The results demonstrated that the reaction rate increased rapidly and then followed a sharp decrease, illustrating an early region of maximum reactivity, the middle part of large reaction rate and a small reactivity in the later stage. Furthermore, there were not any significant reaction rates with gasification temperature rise after 20 min. Finally, ionanofluids with 0.4 wt% nanoparticles showed the elevated stability of the five as-prepared samples.

### 3.7. Specific Heat Capacity

**Figure 11:**
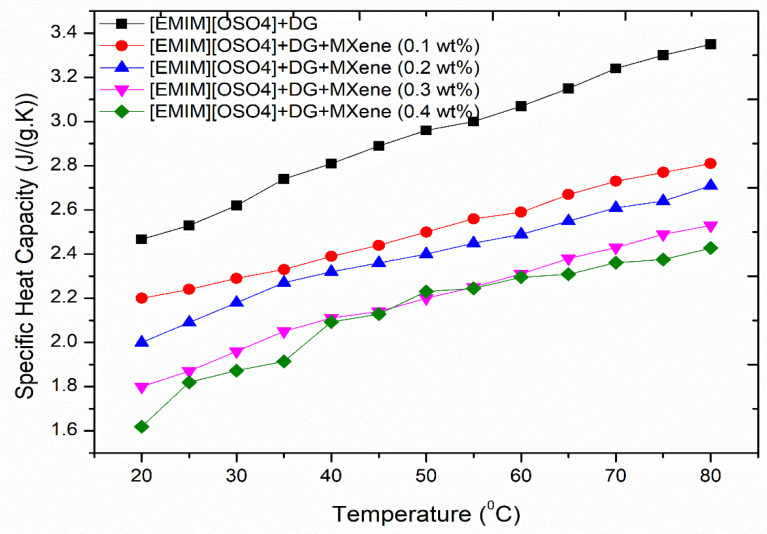
Specific heat capacity of the ionanofluids with temperature increase.

It is a basic property that determines the quantity of heat necessary for raising a substance’s temperature. Figure 11 depicts the specific heat capacity of the proposed fluid at certain temperatures. From the experimental values, a significant increase in the specific heat capacity of the ionanofluids was observed with increase in temperature which is consistent with many of the literature on various types of nanofluids [45,46]. This implies that temperature has a significant effect on the specific heat capacity enhancement of ionanofluids, mainly due to the high surface energy of MXene nanoparticles that can be applied to high-temperature applications where more absorption of heat is desired. More specific heat capacity values are widely favored for the heat transfer fluids to retain more heat from the energy sources. The specific heat capacity of the formulated ionanofluids varied from 1.6 J/g·K to 3.4 J/g·K. The sample without MXene nanoparticles resulted in maximum specific heat capacity illustrating the decrease of specific heat capacity with MXene nanoparticles’ addition.

### 3.8. Heat Transfer Rate Efficiency of the Ionanofluids

The heat transfer efficiency of the as-prepared ionanofluids in turbulent flow conditions can be calculated by the Mouromtseff number [47]. For a flow with specified geometry and velocity, the coolant with the highest Mouromtseff number will lead to a maximum heat transfer rate. Mouromtseff reported the figure of merit (MO) to evaluate the heat transfer capacity of fluids presented in Equation (3).

(3) $$FOM_{MO}=\frac{\rho^{a}k^{b}Cp^{d}}{\mu^{e}}$$

where *ρ*, *k*, *Cp* and *μ* represent the density, thermal conductivity, specific heat capacity and fluid viscosity. Based on the mode of heat transfer and heat transfer correlation, the appropriate values of *a*, *b*, *d* and *e* are calculated by Simons [48] in which this study also pursued. Since the Nusselt number is constant for a fully developed turbulent flow [49], the fluid’s thermal conductivity will play a significant role in determining the heat transfer coefficient of a coolant. Based on the above-discussed constraints, the Mouromtseff number is finally given by Equation (4) which is dependent on the thermophysical properties of the fluids.

(4) $$FOM_{MO}=\frac{\rho^{0.8}k^{0.67}Cp^{0.33}}{\mu^{0.47}}$$

**Figure 12:**
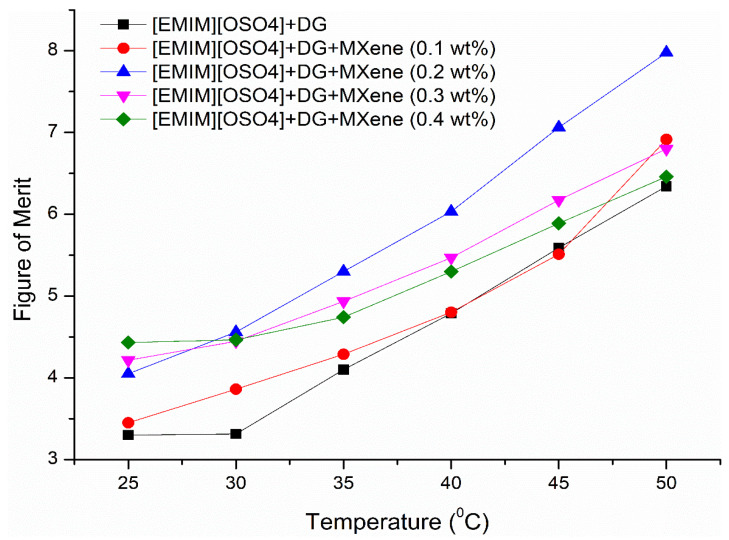
Figure of merit of the prepared ionanofluids for turbulent condition.

Figure 12 illustrates the Figure of Merit (FOM_MO_) as a function of temperature under internal turbulent conditions for the as-prepared ionanofluids. From the graph, it was clear that the Mouromtseff number of the prepared samples was higher than 1, demonstrating that ionanofluids are suitable for utilizing it as heat transfer fluids. Overall, ionanofluids with 0.2 wt% of MXene nanoparticles resulted in a maximum heat transfer rate.

### 3.9. Application of the Ionic Liquid-Based MXene Nanofluid in the PVT System

**Figure 13:**
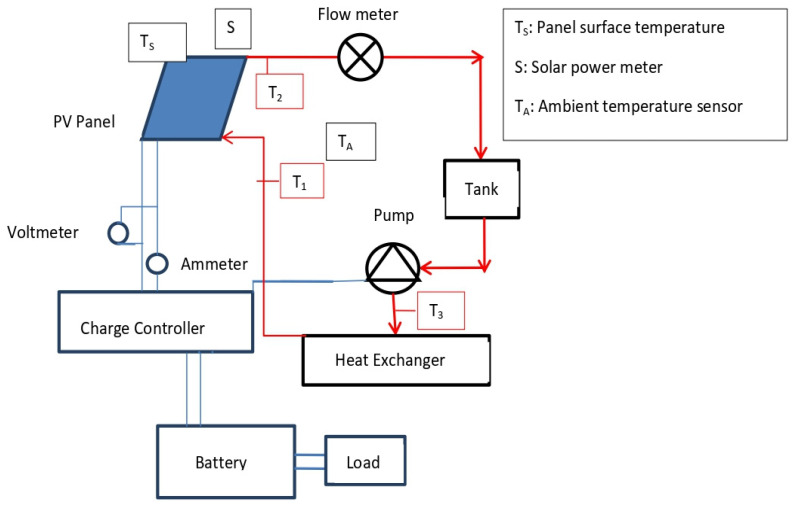
Schematic layout of the experimental setup where T_1_, T_2_ andT_3_ are thermocouples for inlet and outlet temperature measurement of the PV/T system. Thermal circuit is presented in red color and the electric circuit is depicted in blue color.

Researchers employed various active and passive techniques in a hybrid PV thermal system to monitor the PV cell module’s temperature rise. For the past decade, nanofluid has been used a coolant on the PV panels’ rear side to reduce temperatures for better performance, particularly in hot, dry conditions. A numerical analysis is conducted in this study to evaluate the efficiency of the as-prepared ionic liquid-based 2D MXene nanofluid in a photovoltaic/thermal (PV/T) system. In this analysis, Al_2_O_3_/Water and ionic liquid-based MXene nanofluid are used as a coolant in a hybrid PVT system, and their efficiency is numerically compared with water coolant. The problem under investigation is presented in Figure 13.

#### 3.9.1. Numerical Model and Simulation 

For numerical analysis, COMSOL a multiphysics software that works on the Finite Element Method is used. The flow of nanofluid is assumed to be laminar, incompressible, steady and 3D. The dispersion stability of the proposed fluids is considered to be highly stable (no agglomeration). In this study, ionic liquid-based 2D MXene nanofluid containing 0.1, 0.2, 0.3 and 0.4 wt.% nanoparticle concentration is used. With a user-defined feature in COMSOL software, thermal conductivity as a function of concentration and temperature is fitted to the third-order polynomial by regression analysis. Maxwell model is utilized to calculate the thermal conductivity of Al_2_O_3_/water nanofluid where the common thermal conductivity value of 0.611 W/m.K and 40 W/m.K for water and Al_2_O_3_ is used [50] (see Equation (5)).

(5) $$k_{nf}=k_{f}\frac{k_{s}+2k_{f}-2\phi \left(k_{f}+k_{s}\right)}{k_{s}+k_{f}+2\phi \left(k_{f}+k_{s}\right)}$$

For modeling viscosity of the as-prepared ionic liquid-based MXene nanofluid as a function of temperature, regression analysis of experimental data is performed. With User Defined Function (UDF), the Equation is embedded into COMSOL, and the simulation utilizes the user-defined thermal conductivity. Brinkman model is considered for modeling the viscosity of Al_2_O_3_/water nanofluid [51]. The continuity, momentum and energy equations (Equations (6)–(10)) that describe the flow are as follows:

Continuity:

(6) $$\frac{\partial u}{\partial x}+\frac{\partial v}{\partial y}+\frac{\partial w}{\partial z}=0$$

X-momentum:

(7) $$\rho_{nf}\left(u\frac{\partial u}{\partial x}+v\frac{\partial u}{\partial y}+w\frac{\partial u}{\partial z}\right)=\frac{-\partial P}{\partial x}+{µ}_{nf}\left(\frac{\partial^{2}u}{\partial x^{2}}+\frac{\partial^{2}u}{\partial y^{2}}+\frac{\partial^{2}u}{\partial z^{2}}\right)$$

Y-momentum:

(8) $$\rho_{nf}\left(u\frac{\partial v}{\partial x}+v\frac{\partial v}{\partial y}+w\frac{\partial v}{\partial z}\right)=\frac{-\partial P}{\partial y}+{µ}_{nf}\left(\frac{\partial^{2}v}{\partial x^{2}}+\frac{\partial^{2}v}{\partial y^{2}}+\frac{\partial^{2}v}{\partial z^{2}}\right)$$

Z-momentum:

(9) $$\rho_{nf}\left(u\frac{\partial w}{\partial x}+v\frac{\partial w}{\partial y}+w\frac{\partial w}{\partial z}\right)=\frac{-\partial P}{\partial z}+{µ}_{nf}\left(\frac{\partial^{2}w}{\partial x^{2}}+\frac{\partial^{2}w}{\partial y^{2}}+\frac{\partial^{2}w}{\partial z^{2}}\right)$$

Energy equation:

(10) $$\rho_{nf}C_{Pnf}\left(u\frac{\partial T}{\partial x}+v\frac{\partial T}{\partial y}+w\frac{\partial T}{\partial z}\right)=K_{nf}\left(\frac{\partial^{2}T}{\partial x^{2}}+\frac{\partial^{2}T}{\partial y^{2}}+\frac{\partial^{2}T}{\partial z^{2}}\right)$$

It is assumed that nanofluid density ($\rho_{nf}$) and heat capacity ($C_{Pnf}$) are constant, and their values are extracted from the empirical correlation described in the archival literature [52,53] (Equations (11) and (12))

(11) $$\rho_{nf}=\left(1-\phi \right)\rho_{f}+\phi \rho_{s}$$

(12) $$Cp_{nf}=\left(1-\phi \right){\left(C_{P}\right)}_{f}+\phi {\left(C_{P}\right)}_{s}$$

For the proposed PV/T system, energy balance is employed as depicted in Equation (13). This Equation is based on the Sun’s irradiance, panel surface radiation, convection between PV/T and environment, electrical and thermal energy output.

(13) $$G-P_{el}-P_{th}-\overset{´}{Q_{conv}}-\overset{´}{Q_{rad}}=0$$

Equations (14) and (15) are used for modeling the convective and radiative heat transfer of the proposed hybrid PV/T system.

(14) $$-n.\left(-k\nabla T\right)=h\left(T_{amb}-T\right)$$

(15) $$-n.\left(-k\nabla T\right)=\varepsilon \sigma \left(T_{amb}^{4}-T^{4}\right)$$

Equations (16) and (17) are utilized for calculating the electrical power and thermal energy of the studied hybrid PV/T system.

(16) $$P_{el}=V_{oc}*I_{sc}*FF$$

(17) $$P_{th}=mC_{p}\left(T_{out}-T_{in}\right)$$

Using Equations (18) and (19), the hybrid PV/T system’s electrical and thermal efficiency are calculated.

(18) $$\eta_{el}=\frac{P_{el}}{G*A_{c}}$$

(19) $$\eta_{th}=\frac{P_{th}}{G*A_{c}}$$

##### Boundary Conditions

The PV/T system’s side surfaces are assumed to be adiabatic, and continuous heat flux is considered for the solid-fluid interface. Furthermore, the no-slip condition is preferred for the solid boundaries. At the inlet *u* = 0, *v* = *V_o_*, *w* = 0 and *T* = *T_o_*, moreover zero pressure boundary condition is chosen for the outlet. In addition, the bottom panel of the hybrid solar PV/T system is insulated.

##### Meshing and Grid Independency

**Figure 14:**
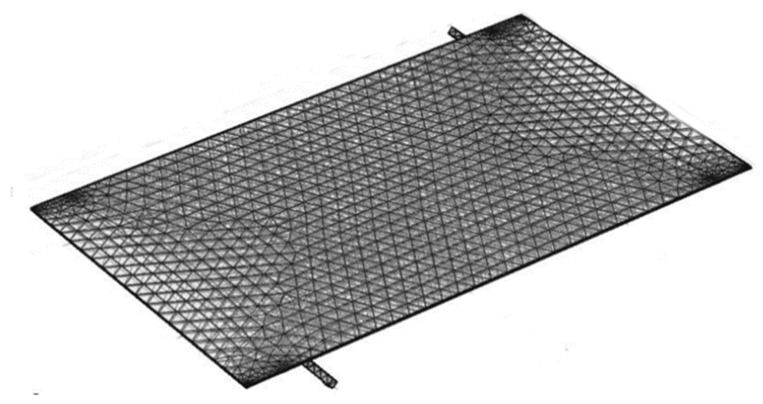
Photovoltaic/thermal (PV/T) model with finite element meshing.

Figure 14 depicts the proposed PVT system’s finite element meshing where the boundaries and subdomains are equipped with triangular and tetrahedral meshes, respectively. The water is chosen as a coolant with various meshes of coarser and finer sizes for independent grid simulation of 1000 W/m^2^ and a mass flow rate of 0.05 kg/s, as seen in Table 4. After the fifth mesh, the temperature value of the cell has not changed. Therefore, this mesh size is chosen for all of the simulations presented in this study.

**Figure 15:**
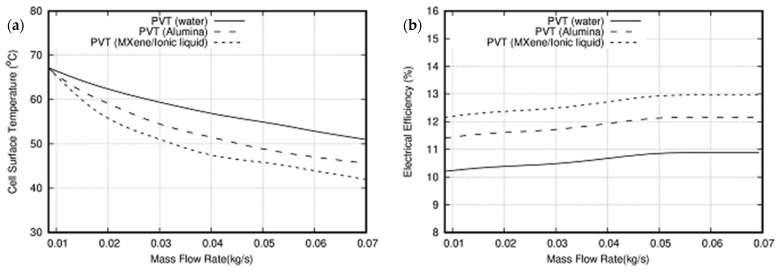

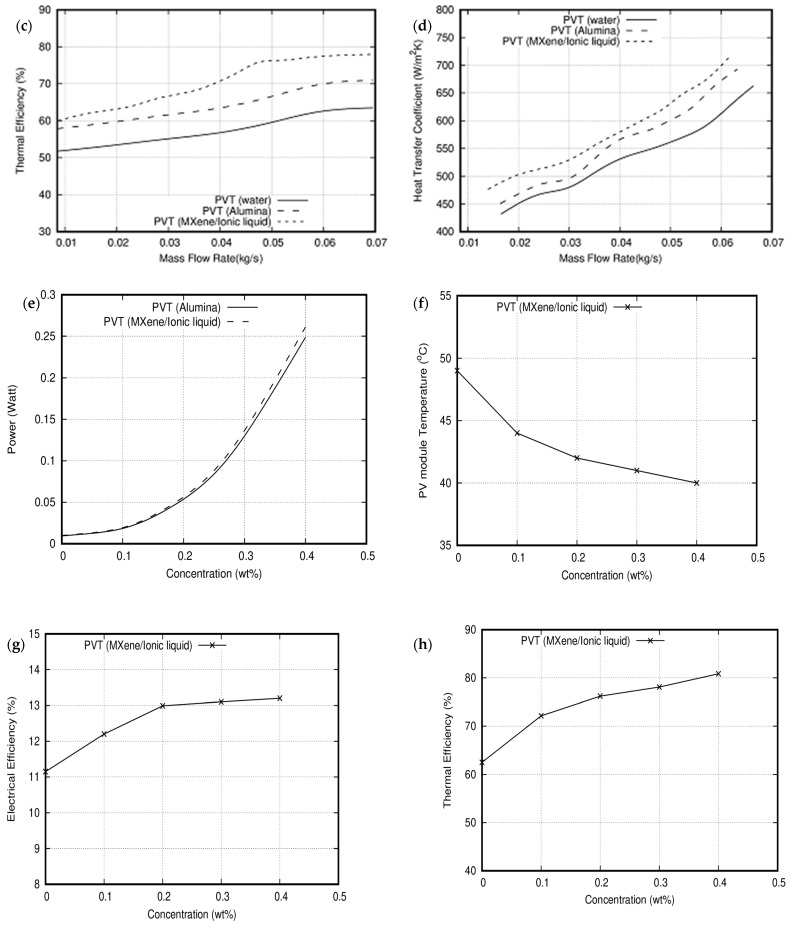
(**a**) PV cell surface temperature, (**b**) electrical efficiency, (**c**) thermal efficiency, (**d**) heat transfer coefficient of the PV/T system as a function of mass flow rate using different coolant, at an irradiance of 1000 W/m^2^ and concentration of 0.2 wt%. (**e**) Pumping power, (**f**) PV module surface temperature, (**g**) electrical efficiency, (**h**) thermal efficiency as a function of concentration at an irradiance of 1000 W/m^2^ and mass flow rate of 0.07 kg/s.

Different coolants were used in this study to maintain the temperature of the PV module within the permitted limit. Figure 15a illustrates a comparison with water, Al_2_O_3_/water and the newly developed ionic liquid-based MXene nanofluid to reveal the effect on PV cell temperature at an irradiance level of 1000 W/m^2^ and at varying mass flow rate. MXene-based fluid has been found to have a higher temperature drop throughout the PV cell, thereby indicating better thermal efficiency. Unfortunately, there was only a small temperature difference on the PV surface at a mass flow rate of less than 0.015 kg/s. Nevertheless, a decreasing pattern is found with a considerable variation attributed to the module’s increased convection rate. As a result, the surface temperature of the module decreased with increased mass flow rate. At a maximum flow rate of 0.07 kg/s, the PV surface temperature due to water, alumina/water and ionic liquid-based MXene nanofluid is 51.5 °C, 45.07 °C and 42.5 °C, respectively.

The comparison between electrical efficiency and mass flow rate variation for the above-mentioned coolants is displayed in Figure 15b. The electrical efficiency increases with mass flow rate, for Ionic liquid-based MXene nanofluid it increases from 12.15% to 13% in the mass flow rate range from 0.01 to 0.07 kg/s. Therefore, utilizing ionic liquid-based MXene nanofluid in the hybrid PV/T system increases the electrical performance by 6.5% relative to alumina nanofluid at a mass flow rate of 0.07 kg/s. Consequently, an 18.7% increase in electrical efficiency is obtained relative to water as a coolant at 0.07 kg/s mass flow rate.

Variation of thermal efficiency with respect to the mass flow rate of the three coolants is presented in Figure 15c. From the graph, increased mass flow rates improve the thermal efficiency of the PV/T system regardless of the coolant type. The maximum thermal efficiency of 63.3%, 71.18% and 77.5% is obtained for water, alumina and ionic liquid-based MXene nanofluid, respectively at a mass flow rate of 0.07 kg/s. Results indicate that ionic liquid-based MXene nanofluid performs better than alumina/water nanofluids and demonstrates a strong heat transfer potential. In contrast to aluminum/water nanofluid, MXene-based fluid improved the thermal performance of the studied PV/T system by 10.28%.

Figure 15d depicts the as-prepared sample’s heat transfer coefficient with respect to mass flow rate. The results illustrated that a rise in the mass flow rate contributes to an increase in the heat transfer coefficient irrespective of the fluid type used in this analysis. A maximum heat transfer coefficient of 6% for ionic liquid-based MXene nanofluid compared to alumina/water nanofluid is obtained at 0.06 kg/s.

Figure 15e illustrates the pumping power required for nanofluid circulation with the concentration of nanoparticles in the base fluid. Results demonstrate that the power needed for pumping has a negligible effect if the nanofluid is changed to ionic liquid-based MXene nanofluid in lieu of Alumina/water. This change is slightly remarkable when the concentration is 0.4 wt%. Therefore, it is concluded that pumping power has a negligible effect on PV/T performance. Figure 15f shows the PV module temperature with nanoparticles’ concentration at a flow rate of 0.07 kg/s and irradiance of 1000 W/m^2^. It is clear from the figure as the concentration increases the PV module temperature decreases. Further, the percentage of temperature drop from 0.1% to 0.4% concentration is 10% which is itself remarkable and solely due to the enhancement of thermal conductivity.

Figure 15g,h show the electrical and thermal efficiency with concentration, respectively, at a flow rate of 0.07 kg/s and irradiance of 1000 W/m^2^. Both the efficiencies improve with an increase in concentration; electrical efficiency improves around 8.2% when concentration increases from 0.1 to 0.4 wt%. Simultaneously, thermal efficiency has an improvement of 10.9% in the concentration range of 0.1 to 0.4 wt%.

## 4. Conclusions

Novel nanofluids containing MXene nanoparticles in diethylene glycol/[C_8_SO_4_] ionic liquid mixture were formulated via the two-step method, which was subjected to various temperature ranges for analyzing its thermophysical and rheological properties. [C_8_SO_4_] and its MXene dissolved nanofluids attained good thermal stability because of their initial thermal decomposition temperature of as high as 316 °C. The thermal conductivity of the ionanofluids increased linearly with the rise in temperature which ranged from 25 to 80 °C, whereas the viscosity and density of the ionanofluids decreased drastically especially in the range of 35 to 80 °C. Results demonstrate that the thermal conductivity and thermal stability of the ionanofluids could be improved by increasing the MXene nanoparticle concentration and temperature. The rheological behavior of the ionanofluids revealed that the proposed fluids were Newtonian at the tested temperatures. According to the Mouromtseff number and thermal properties analysis, of the five tested fluids, the optimum thermal efficiency was obtained for 0.2 wt% mass concentration. From the numerical simulation results, it is clear that the PV module temperature decreases with increase in the ionanofluid concentration, further the percentage of temperature drop from 0.1% to 0.4 wt% concentration is 10% which is itself remarkable. The final results demonstrated that for the maximum flow rate (0.07 kg/s) thermal efficiency, the studied fluid’s electrical efficiency and heat transfer coefficient increased by 78.5%, 18.7% and 6%, respectively. Therefore, Diethylene glycol/[C_8_SO_4_] and MXene dispersed ionanofluid have a greater heat transfer potential than the conventional heat transfer fluids which makes them suitable for advanced heat transfer fluids in high and medium temperature applications.

## Figures and Tables

**Table 1:** Recent research on MXene and its applications.

References	Material	Additives	Applications	Findings
Lin et al. [13]	Ti_3_C_2_	PEG4000	Solar energy conversion	Superior light absorption capacity at 808 nm in UV–Vis-NIR region Thermal stability of PEG improved by 40 °C with the addition of MXene
Lu et al. [14]	Ti_3_C_2_	PEG	Thermal energy storage	Maximum thermal conductivity of 2.052 W/m·K was achieved Inclusion of MXene decreased the crystalline regions of PEG
Wu et al. [15]	Ti_3_C_2_	SA SO SC SP	Micro-Super capacitor	With the addition of ascorbate ions the interlayer gap of MXene nanosheets increased
Zhang et al. [16]	Ti_3_C_2_	Dimethyl sulfoxide Absolute ethanol Deionized water	Memristor	Clear resistance transforms between the low resistance state and high resistance state was observed MXene enhanced the retention characteristics of the proposed device
Xiao et al. [17]	Ti_2_C Ti_2_CC_2_ Ti_2_CO_2_ Ti_2_CS_2_	Sodium-ion	Na-ion Batteries	Stable adsorption of sodium ions on the monolayers of MXene Maximum interlayer distance and energy storage capacity of 20 Å and 536.84 mA·h·g^−1^ was obtained
Rong et al. [18]	Ti_3_C_2_	CdS	Photocatalysis	MXene enhanced the transfer of electrons and electron-hole separation
Rajavel et al. [19]	Ti_3_C_2_	PVDF	Heat dissipation	Maximum thermal and electrical conductivity of 0.767 W/m·K and 0.98 S/m was obtained

**Table 2:** Proportion of nanoparticles, ionic liquids and diethylene glycol.

Sample	Weight (%)	Total Mass (gram)	Mass ± 0.001 g		
			[C_8_SO_4_]	Diethylene Glycol	MXene Nanoparticle
[C_8_SO_4_]+DEG	0	50	5	45	0
[C_8_SO_4_]+DEG+MXene	0.1	50	4.995	44.955	0.05
[C_8_SO_4_]+DEG+MXene	0.2	50	4.990	44.91	0.1
[C_8_SO_4_]+DEG+MXene	0.3	50	4.985	44.865	0.15
[C_8_SO_4_]+DEG+MXene	0.4	50	4.980	44.82	0.2

**Table 3:** Uncertainty of different quantities for the present study.

Device	Quantity	Accuracy	Maximum Uncertainty (In Experiments)
Thermal property analyzer (Tempos)	Thermal conductivity	±10%	0.04 W/m·K
Density meter	Density	±0.001 g/cm^3^	0.003 g/cm^3^
Rheometer	Viscosity	±1%	1.15 mPa·s
	Rheology	±1%	0.06 Pa
Differential Scanning Calorimetry	Specific heat capacity	±2%	0.08 J/g·K
Thermogravimetric Analyzer	Thermal stability	±0.02%	0.09%
FTIR Spectrometer	Light transmittance	±1%	0.22%

**Table 4:** Grid independency test.

Serial No.	Size of Mesh (No. of Elements)	Temperature of Cell (°C)
1	2.5 × 10^5^	42.341
2	4 × 10^5^	43.872
3	6 × 10^5^	44.003
4	8 × 10^5^	44.118
5	1.5 × 10^6^	45.200
6	3.5 × 10^6^	45.201

**Figure A1:**
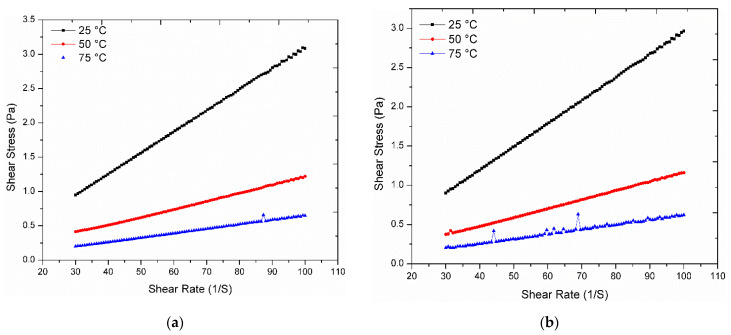

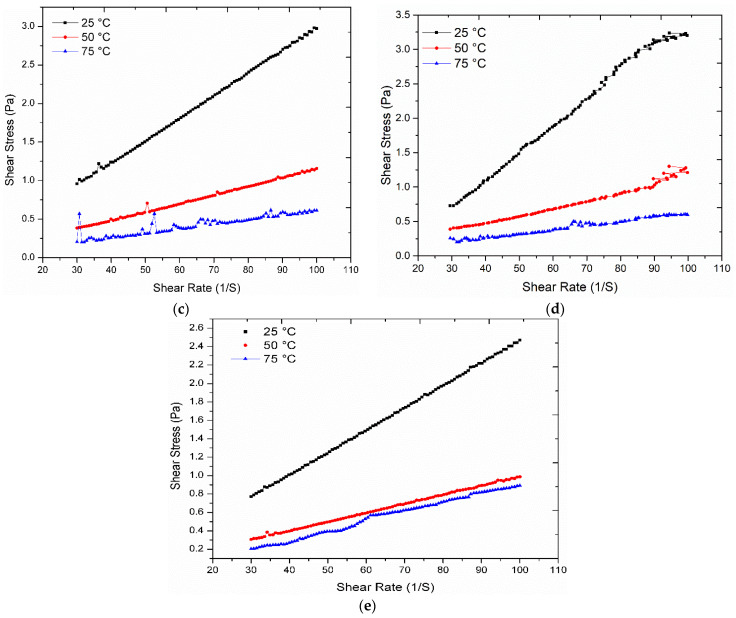
Viscosity versus shear rate for (**a**) [C_8_SO_4_]+DEG, (**b**) [C_8_SO_4_]+DEG+0.1 wt% MXene, (**c**) [C_8_SO_4_]+DEG+ 0.2 wt% MXene, (**d**) [C_8_SO_4_]+DEG+ 0.3 wt% MXene, (**e**) [C_8_SO_4_]+DEG+ 0.4 wt% MXene.

## Data Availability

The data presented in this study are available on request from the corresponding author. The data are not publicly available due to privacy issue.

## Supplementary Materials

The following are available online at https://www.mdpi.com/2079-4991/11/2/320/s1, In the supplementary file, the uncertainty calculation for the measured experimental quantities via GUM (Guide to the expression of Uncertainty in Measurements) is included.

## Nomenclature

[C_8_SO_4_]	1-ethyl-3-methyl imidazolium octylsulfate
[EMIM][BF_4_]	1-methyl-3-ethylimidazolium tetrafluoroborate
[BMIM][BF_4_]	1-methyl-3-butylimidazolium tetraflouoroborate
[BMIM][Br]	1-butyl-3-methylimidazolium bromide
[DMPI][FSI]	1,2-dimethyl-3-propylimidazolium-bis (trifluorosulfonyl) imide
[C4MIM][NTf_2_]	1-butyl-3-methylimidazolium bis(trifluoromethylsulfonyl)imide
[P_14,6,6,6_][RO]	Trihexyl(tetradecyl)phosphonium carboxylate
PVT	Photovoltaic Thermal System
MXene	Ti_3_C_2_
DEG	Diethylene Glycol
HTF	Heat Transfer Fluid
PEG	Polyethylene Glycol
PVDF	Polyvinylidene Fluoride
SA	Sodium Ascorbate
SO	Sodium Oxalate
SC	Sodium Citrate
SP	Sodium Phosphate
Ra	Rayleigh Number
Al_2_O_3_	Alumina
SiO_2_	Silicon dioxide
Ra	Rayleigh number
ϕ	Concentration
S/m	Siemens per meter
γ-Al_2_O_3_	Gamma Alumina
Pa	Pascal
Nb	Niobium
Ta	Tantalum
Ti	Titanium
